# Safety of Novel Microbes for Human Consumption: Practical Examples of Assessment in the European Union

**DOI:** 10.3389/fmicb.2017.01725

**Published:** 2017-09-12

**Authors:** Theodor Brodmann, Akihito Endo, Miguel Gueimonde, Gabriel Vinderola, Wolfgang Kneifel, Willem M. de Vos, Seppo Salminen, Carlos Gómez-Gallego

**Affiliations:** ^1^Department of Food Sciences and Technology, University of Natural Resources and Life Science Vienna Vienna, Austria; ^2^Department of Food and Cosmetic Science, Tokyo University of Agriculture Hokkaido, Japan; ^3^Instituto de Productos Lácteos de Asturias, Spanish Higher Research Council Villaviciosa, Spain; ^4^Instituto de Lactología Industrial (UNL-CONICET), National University of the Litoral Santa Fe, Argentina; ^5^Laboratory of Microbiology, Wageningen University and Research Wageningen, Netherlands; ^6^Immunobiology Research Program, Research Programs Unit, Faculty of Medicine, University of Helsinki Helsinki, Finland; ^7^Functional Foods Forum, Faculty of Medicine, University of Turku Turku, Finland

**Keywords:** novel microbes, safety, *Bacteroides xylanisolvens*, *Akkermansia muciniphila*, fructophilic lactic acid bacteria, *Faecalibacterium prausnitzii*

## Abstract

Novel microbes are either newly isolated genera and species from natural sources or bacterial strains derived from existing bacteria. Novel microbes are gaining increasing attention for the general aims to preserve and modify foods and to modulate gut microbiota. The use of novel microbes to improve health outcomes is of particular interest because growing evidence points to the importance of gut microbiota in human health. As well, some recently isolated microorganisms have promise for use as probiotics, although in-depth assessment of their safety is necessary. Recent examples of microorganisms calling for more detailed evaluation include *Bacteroides xylanisolvens, Akkermansia muciniphila*, fructophilic lactic acid bacteria (FLAB), and *Faecalibacterium prausnitzii*. This paper discusses each candidate's safety evaluation for novel food or novel food ingredient approval according to European Union (EU) regulations. The factors evaluated include their beneficial properties, antibiotic resistance profiling, history of safe use (if available), publication of the genomic sequence, toxicological studies in agreement with novel food regulations, and the qualified presumptions of safety. Sufficient evidences have made possible to support and authorize the use of heat-inactivated *B. xylanisolvens* in the European Union. In the case of *A. muciniphila*, the discussion focuses on earlier safety studies and the strain's suitability. FLAB are also subjected to standard safety assessments, which, along with their proximity to lactic acid bacteria generally considered to be safe, may lead to novel food authorization in the future. Further research with *F. prausnitzii* will increase knowledge about its safety and probiotic properties and may lead to its future use as novel food. Upcoming changes in EUU Regulation 2015/2283 on novel food will facilitate the authorization of future novel products and might increase the presence of novel microbes in the food market.

## Introduction

Since ancient times, microorganisms have constituted an essential part of human nutrition and been consumed through naturally microbially fermented products containing viable bacteria, such as fermented honey, fruits, berries, and their juices and fermented products of animal origin. Without even knowing of the existence of bacteria, humans historically used them to initiate many food production processes (Selhub et al., 2014). Today, an enormous variety of fermented foods and beverages exists and provides approximately one-third of the human diet globally (Borresen et al., 2012). Certain fermented foods containing significant amounts of viable microbes, especially dairy products and fermented vegetables, are part of daily consumption by millions of people around the globe.

Studies involving new analytical technologies have generated new insights into the human and animal guts and yielded new isolates and candidates that perform special functions in this complex intestinal environment (Marchesi et al., 2016). The composition of human gut microbiota plays an important role in homeostasis and gut functionality, which are also strongly influenced by age, diet, environmental factors, disorders, diseases, and therapies. In recent decades, probiotic microorganisms have been identified as efficient modulators of intestinal microbial balance (Gómez-Gallego and Salminen, 2016). Probiotics are defined as live microorganisms that confer a health benefit to the host when administered in adequate amounts (Cammarota et al., 2014; Hill et al., 2014). These health benefits are provided through relatively common mechanisms found in the vast majority of the investigated probiotics, such as regulation of the intestinal transit, competitive exclusion of pathogens, and production of specific short chain fatty acids (SCFA). In addition, frequently observed mechanisms mostly found on a species level include vitamin synthesis, enzymatic activity, gut barrier reinforcement, and neutralization of carcinogens. The existence of a group of basic benefits shared by strains of the same species in a non-strain specific manner supports the concept of core benefits for specific species. Moreover, mechanisms described exclusively on the individual strain level provide immunological, neurological, and endocrinological effects. Such benefits in general support a healthy digestive tract and immune system (Aureli et al., 2011; Gómez-Gallego and Salminen, 2016).

Traditionally fermented foods, especially fermented dairy products, usually contain viable bacteria. The consumption of such foods is widely connected with the reduction of certain disease types (e.g., childhood obesity, type-2 diabetes, and cardiovascular diseases), improved body composition, and weight loss in adults during energy restriction (Thorning et al., 2016). The International Scientific Association for Probiotics and Prebiotics (ISAPP) panel acknowledged convincing results but also emphasized the difficulty of verifying whether the beneficial results originate from the food matrices, the (viable) microorganisms, or a combination of both. Additionally, traditional fermented foods do not qualify for the term *probiotic* because they may contain varying amounts and changing microbial species over time, and their microbiological composition is not completely known or controlled. A relevant example is traditional Kefir. The ISAPP, therefore, has proposed the term “containing live and active bacterial cultures” for traditional fermented foods (Hill et al., 2014).

Probiotics relevant to the human gut microbiota mainly belong to the genera *Lactobacillus* and *Bifidobacterium*, but several other microbial candidates, such as *Akkermansia muciniphila* and *Faecalibacterium prausnitzii*, could play roles in future probiotic products. Although, commensal gut microbes can provide beneficial health effects, the ISAPP panel suggested conducting strain-by-strain assessment until sufficient research data are available to grant probiotic status on the species level (Hill et al., 2014).

## European union regulations of microorganisms intentionally added to food

The European Union (EU) is a single-market system consisting of 28 member states and the four members of the European Free Trade Association (Iceland, Liechtenstein, Norway, and Switzerland), which are required to follow European legislation as part of the EU single market. The European Food Safety Authority (EFSA) has the mandate to assess and communicate all risks associated with the food chain in the EU. The EFSA Scientific Committees and Panels are responsible for providing scientific opinions in response to requests from the European Commission, European Parliament, and EU member states. Before the EFSA was established, risk assessment was performed by Scientific Committees assisting the Directorate General for Health and Consumers (DG SANCO). DG SANCO, re-named to DG SANTE, continues to perform a crucial regulatory role regarding microorganisms in food and feed. Standing Committees representing member states, and their interests have strong influence on the work of DG SANTE (von Wright, 2012).

Amid globalization and ongoing technical progress, the number of new food products in the EU has increased significantly, and food safety has become a growing concern. A uniform regulation for novel food was needed to ensure the protection of both European consumers and the market. The introduction of novel foods is governed by Regulation 285/97/EC (European Commission, 1997b) and Regulation 2015/2283 (European Commission, 2015), covering all foods not been used in the EU before 15 May 1997. Under Regulation 2015/2283, the EFSA, rather than competent national authorities, will perform the scientific risk assessment for novel foods; the system of individual authorization is replaced by a system of generic authorization; and the Commission will manage all applicants' files and prepares proposals for the authorization of novel foods found to be safe (European Commission, 2015). To speed decision-making, the EFSA is required to issue its opinion within 9 months of the date of receipt of a valid application.

The guidance lists general requirements for every application: (1) a description; (2) compositional data; (3) production process; (4) specifications; (5) proposed uses and use levels; and (6) anticipated intake of the novel food. Applicants also have to provide information about the absorption, distribution, metabolism, excretion, nutrition, toxicology, allergenicity, and history of use of novel foods or their source. In addition, all the novel foods that belong to the group of “foods consisting of, isolated from or produced from micro-organisms, fungi or algae” must provide the following information (EFSA Panel on Dietetic Products, 2016): (1) scientific (Latin) name (family, genus, species, strain) according to the international codes of nomenclature; (2) synonyms used interchangeably with the preferred scientific name; (3) for bacteria and yeasts (unicellular organisms), verification of the species and strain identity according to internationally accepted methods; (4) origin of the organism; and (5) if available, deposition in an officially recognized culture collection with an access number.

### Qualified presumption of safety

EFSA introduced the concept of qualified presumption of safety (QPS) to establish a generic risk assessment approach for biological agents, with the goal to simplify and harmonize assessment of notified biological agents across the EFSA's various Scientific Panels and Units. The QPS approach also enables more focused application of the available resources to agents with higher risk potential (Leuschner et al., 2010), simplifying the assessment of low-risk microorganisms. The QPS concept is intended to establish a safety assessment for microorganisms used in feed and food production. If a specific strain notified for market authorization can unambiguously be connected to a taxonomic unit on the QPS list, the necessary safety assessment steps, if any, are indicated in the list for that taxonomic unit.

Bacteria with a previous history of safe use are usually included on the QPS list. The EFSA's Panel on Biological Hazards assesses the QPS status of a strain and declares that the assigned microorganism displays either no safety concerns or minor concerns defined and addressed with qualifications as expressed in the QPS list. Microorganisms on the QPS list, therefore, need no exhaustive safety assessment except for those specified in the QPS list.

The assessment for QPS suitability is well structured and relies on the following criteria (EFSA, 2007):

- Taxonomic unit: This is the most crucial part of the assessment procedure. The genus and all known species, including type species, are listed and described according to their main characteristics. The evaluated taxonomic unit must be unambiguously defined. If the assessed bacterial agent cannot be connected to any known species, it will not be recommended for the QPS list. QPS status is also denied if the taxonomic identification is insufficient.
- Body of knowledge: This should be related to the history of use for the assessed bacterial agent, the field of application, the existence of sufficient scientific literature about the microorganism, and the possibility to avoid potential adverse effects for humans, livestock, and the wider environment.
- Possible pathogenicity: The bacterial agent's lack of pathogenic and virulence properties is confirmed, and the findings must be supported by clinical data and scientific literature.
- Description of end use: This includes the presence and viability of the microorganism in the final product.

Most bacteria recommended for the QPS list belong to the group of Gram-positive non-sporulating bacteria. This group includes many inhabitants of the digestive tract with long histories in food and feed production. For this group, the EFSA's Scientific Committee applies a generic qualification for all taxonomic units on the list, requiring that all strains not carry any transferable antimicrobial resistance unless the final product contains no viable cells (Leuschner et al., 2010). The number of recommended Gram-negative bacteria is very low, with only one, *Gluconobacter oxydans*, on the QPS list. Many members have a long history of safe use, but safety concerns cannot be excluded. *E. coli* strain Nissle 1917, for example, has long been used as a probiotic but can cause a variety of diseases and exhibits versatile virulence mechanisms. Nevertheless, opportunistic bacteria may be placed on the QPS list with additional qualifications, but pathogenic and toxin-producing bacteria are not included.

Microorganisms whose safety properties are not fully understood are subjected to safety assessments. Such assessments need to include a distinct taxonomic classification on the species or the strain level and a comprehensive strain characterization, including whole-genome sequence analysis, to identify potential virulence and antibiotic resistance genes and their horizontal transfer capabilities. However, in the case of new and recently discovered microorganisms from previously unknown microbial groups, the availability of genome sequences might not be enough to identify potential virulence or antibiotic resistance genes. These often require comparison with available data, so functional studies on these microorganisms are needed. For accurate safety evaluation, the food business operator should include the number and the viability of microorganisms in the final product by.

The QPS approach was inspired and influenced by the American Generally Recognized As Safe (GRAS) concept, but differences exist. QPS is defined as an “assumption based on reasonable evidence” (H. C. P. Directorate-General, 2003), providing a helpful assessment tool for the EFSA but, in contrast to GRAS, offers no legal status. The EFSA is responsible for providing the burden of proof, while GRAS lays the responsibility on the food business operator. GRAS, though, requires safety assessment by independent experts to the degree that another panel of independent experts would reach the same conclusions. The QPS assessment's strong focus on the absence of acquired antibiotic resistances and virulence factors is another difference between the two concepts.

## Microorganisms authorized as novel food in the EU

### Leuconostoc mesenteroides

In January 2001, the European Commission authorized market placement of a dextran preparation produced by *L. mesenteroides* as a novel food ingredient in bakery products. Puracor NV (Groot-Bijgaarden, Belgium) filed the application, and the Belgian competent authorities performed the initial assessment. The Scientific Committee for Food found that the dextran preparation by *L. mesenteroides* was safe for human consumption up to 5% in bakery products. Dextran was identified as a highly digestible bakery ingredient with similar nutritional properties as starch (European Commission, 2001).

*L. mesenteroides* is a Gram-positive, non-sporulating, coccoid-shaped bacterium in the order Lactobacillales. The organism is often found on the surfaces of various plant parts and is responsible for the fermentation of white cabbage into sauerkraut. *L. mesenteroides* can be also found in artisanal and industrial cheeses, possibly contributing to their safety for long-term consumption (Cibik et al., 2000; D'Angelo et al., 2017). The species can metabolize a broad range of sugars; in particular, sucrose is used to build dextran, and certain strains can produce bacteriocins with anti-listerial activity (de Paula et al., 2015).

### *Bacillus subtilis* natto

In April 2009, the European Commission allowed placing Vitamin K2 (menaquinone), produced by *B. subtilis* natto, in the market as a novel food ingredient under Regulation (EC) No 258/97. NattoPharma (Oslo, Norway) requested that the Irish competent authorities place *B. subtilis* natto derived Vitamin K2 in the market as a novel food ingredient to be used in foods which served particular nutritional uses and which had added vitamins and minerals (European Commission, 2009). In an initial report, the Irish competent authorities required an additional assessment, so the EFSA was requested to conduct an extended assessment. The Scientific Panel on Dietetic Products, Nutrition, and Allergies concluded that *B. subtilis* natto is a safe source of vitamin K2.

*B. subtilis* natto is a Gram-positive, aerobic member of the spore-forming genus *Bacillus*. This species, discovered in Japan in 1906, is responsible for production of natto, a sticky fermented soy beans. Natto, produced by *B. subtilis* natto, has long been used in the Japanese diet, so it gained Foods for Specified Health Use approval based on its health benefits from the Japanese Ministry of Health, Labor, and Welfare. *B. subtilis* has a recognized history of safe use, so the EFSA granted it QPS status (EFSA Panel on Biological Hazards, 2010).

### Clostridium butyricum

In December 2014, the European Commission authorized placing in the market *C. butyricum* CBM 588 as a novel food ingredient according to Regulation (EC) No 258/97 (European Commission, 2014). The British competent authorities performed the initial assessment after Miyarisan Pharmaceutical Co. Ltd. (Tokyo, Japan) requested using *C. butyricum* on the market as a novel food ingredient used in food supplements. Some beneficial effects reported for *C. butyricum* are related to the production of SCFA and its role in lipid metabolism (Zhao et al., 2014; Shang et al., 2016). No additional assessment was necessary because further explanations provided by the applicant addressed the objections raised by member states. *C. butyricum* was authorized as novel food ingredient in food supplements at a maximum dose of 1.35 × 10^8^ CFU per day. Intervention studies revealed the health benefits of the strain, including prevention of pouchitis in patients with ulcerative colitis and decreased incidence of side effects in patients receiving therapy for the eradication of *Helicobacter pylori* (Shimbo et al., 2005; Yasueda et al., 2016). An antibiotic resistance profile, a study for genes encoding toxins, and an animal study were conducted to ensure the safety of the strain (Isa et al., 2016). The authorized strain is a Gram-positive, spore-forming, obligate aerobic, non-pathogenic, non-genetically modified organism. The product is characterized as a white or pale gray tablet with a specific odor and sweet taste (European Commission, 2014).

### Bacteroides xylanisolvens

The most recent authorization for bacteria according to Regulation (EC) No 258/97 was granted in 2015. The European Commission approved placing pasteurized milk products fermented with *B. xylanisolvens* DSM 23964 in the market as a novel food in a heat-treated, non-viable form. Details concerning *B. xylanisolvens* are discussed in the following section.

## Novel microbes: details and background regarding safety

### *Bacteroides xylanisolvens* DSM 23964

In 2015, pasteurized milk products fermented with *B. xylanisolvens* DSM 23964 were approved as a novel food under Novel Food Regulation No 258/97. Usage of this specific strain was restricted as the starter culture in the fermentation of pasteurized milk products. Only heat-treated and therefore inactivated cells of *B. xylanisolvens* were allowed in the final product (EFSA Panel on Dietetic Products, Nutrition and Allergies, 2015). In addition to the novel food evaluation, the EFSA automatically assessed *B. xylanisolvens* for admission to the QPS list. Interestingly, the EFSA revealed that the available information was not sufficient to include it in the QPS list.

The genus *Bacteroides* is a main inhabitant of the human colon, on average accounting for ~30% of intestinal microbiota, although large inter-individual variability exists (Sears, 2005). *Bacteroides* spp. is a Gram-negative, obligately anaerobic, bile resistant, non-spore forming rod originally isolated from human stool. This genus can have commensal attributes within the human body but can also promote the development of different diseases. *Bacteroides xylanisolvens* plays a major part in the fermentation and degradation of xylan and other plant fibers (Chassard et al., 2008). *Bacteroides* start colonization of the gut in the newborn child ~10 days after birth, (Gregory et al., 2015). Polysaccharides in the human diet are an important nutrition source for gut commensals, including *Bacteroides* spp. They can be metabolized into short chain fatty acids and branched chain fatty acids that are reabsorbed through the large intestine and provide an essential part of the host's daily required energy (Hooper et al., 2002).

The levels of Bacteroidetes and Firmicutes seem to be connected to obesity. Obese individuals show lower levels of Bacteroidetes and higher levels of Firmicutes in the gastro-intestinal tract. Turnbaugh and his colleagues (Turnbaugh et al., 2006) suggested that a higher Bacteroidetes/Firmicutes ratio can extract more energy from a given diet and that increased Bacteroidetes levels in the gut led to weight loss in people with obesity.

While investigating xylan-degrading microorganisms in the human gastro-intestinal tract, Chassard et al. (2008) isolated 6 xylanolytic, Gram-negative anaerobic rods. Subsequent 16S rRNA analysis revealed that the obtained strains belonged to the genus *Bacteroides*, and after proper analysis, the 6 strains were considered to be novel species, and the researchers proposed the name *B. xylanisolvens* based on their xylan-degrading properties. The main characteristics of the designated type strain are shown in Table 1.

**Table 1:** General characteristics of *Bacteroides xylanisolvens*.

**Phylum**	**Class**	**Order**	**Family**	**Genus**	**Species**
Bacteroidetes	Bacteroidetes	Bacteroidales	Bacteroidaceae	*Bacteroides*	*Bacteroides xylanisolvens*
Type strain	DSM 188367 (XB1AT)				
Biosafety level	1				
Origin	Faecal sample from a healthy adult volunteer				
**CHARACTERISTICS**					
Morphology	Pleomorphic, Gram-negative, rod-shaped bacterium with rounded ends,				
	1.8–2.5 μm (length), 0.2–0.3 μm (width), no endospores, single or paired cells, no filaments				
Genome	Size: 6,059 kb, ORF's: 4,922, GC content: 41%				
Physiology	Chemo-organoheterotroph, mesophilic (25–42°C—optimum 38°C), pH optimum 6.8, obligate anaerobe				
	Indole negative, catalase negative, xylan positive, D-mannitol positive				
	No starch utilization				

The German Federal Institute for Occupational Safety in Health (*Bundesanstalt für Arbeitsschutz und Arbeitsmedizin*, BAuA) assessed the safety of *B. xylanisolvens* as a working material (BAuA, 2011). Due to the lack of pathogenicity and diseases, the agency classified the microorganism in the lowest risk group. The recently approved strain *B. xylanisolvens DSM 23964* was first analyzed by Ulsemer and colleagues in 2011 (Ulsemer et al., 2012a,b). They isolated this strain from human feces and performed several analytical techniques to verify that it was new. Biochemical characteristics (no catalase activity, no indole production, and incapable of degrading starch), phylogenetic analysis, and DNA-DNA hybridization classified the strain as *B. xylanisolvens*. So far, the performed analyses have established no differences between the new strain and the type strain. Finally, the Random Amplification of Polymorphic DNA profile revealed significant differences, confirming the existence of the new strain *B. xylanisolvens* DSM 23964.

#### Scientific opinion of the EFSA panel on *Bacteroides xylanisolvens* DSM 23964

Following a request from the European Commission, the EFSA Panel on Dietetic Products, Nutrition and Allergies carried out additional assessment for pasteurized milk products fermented with *B*. *xylanisolvens* DSM 23964 as a novel food under Regulation (EC) No 258/97 (European Commission, 1997b). This strain has no history of use in the food industry, and no strain in the genus *Bacteroides* has a proven history of use in food production. The novel food status is related to low-fat and skimmed milk products fermented with *B. xylanisolvens* DSM 23964 as the starter culture. After fermentation, the product is heat treated for 1 h at 75°C, and no viable cells of *B. xylanisolvens* are found in the final product.

The relevant phenotypic and genotypic data were provided by the applicant, and the EFSA Panel considered this information regarding the characterization of *B. xylanisolvens* DSM 23964 to be sufficient. The production process was clearly described in the application, and a study to guarantee the effectiveness of the heat treatment was conducted. Consequently, the EFSA Panel deemed the applied techniques to be standardized across the dairy industry, considered that they are sufficiently described, and identified no safety concerns (EFSA Panel on Dietetic Products, Nutrition and Allergies, 2015).

The applicant also provided the anticipated intake of the product. The novel food would be marketed in liquid and semi-liquid forms in fermented, low-fat milk and skimmed milk products (fermented milk, buttermilk, yogurt, and yogurt drinks) or as a spray-dried powder (the fillings and coatings of cereals, cereal bars, fruits, and nuts). Due to a lack of European data, the applicant used consumption data from the United States. A conservative scenario was used to estimate the intake, proposing that the applicant's products would replace all existing products (e.g., yogurt and buttermilk; EFSA Panel on Dietetic Products, Nutrition and Allergies, 2015).

The applicant provided compositional data for spray-dried skimmed milk cultured with *B. xylanisolvens* DSM 23964, including an overview of relevant macronutrients. The applicant also supplied an overview of the vitamin B2, vitamin B12, free lysine, and furosine (as a marker for Maillard reaction) content to ensure that the heat treatment had no impact on these vitamins. Although, lactose content was missing from the analysis (a comparable amount to traditional products was assumed), the EFSA Panel considered consumption of the novel food to not be nutritionally disadvantageous.

The applicant also provided toxicological information about the novel food, including a study on an intraperitoneal abscess formation model in mice (Ulsemer et al., 2012b). Abscess formation is a relevant pathology because some species in the genus *Bacteroides*, such as *B. fragilis*, can induce abscesses on multiple sites in the body. *B. xylanisolvens* DSM 23964 was administered in varying doses, but none of the mice developed abscesses. Human studies were also supplied to the EFSA (Ulsemer et al., 2012a,c).

Although, the applicant did not provide material regarding the allergenicity of the product, the EFSA panel judged that it would be unlikely that the allergenic potential would differ from that of other fermented dairy products. The EFSA itself had previously considered the effect of heat treatment on the allergenicity of milk (EFSA Panel on Dietetic Products EFSA Panel on Dietetic Products, Nutrition and Allergies, 2014).

#### QPS evaluation of *B. xylanisolvens*

When the EFSA receives an application for a novel bacterial strain or a food product containing a novel strain, it conducts a mandatory, in-depth evaluation based on the QPS scheme. Consequently, after the EFSA Panel on Dietetic Products, Nutrition, and Allergies was asked to perform an additional assessment on *B. xylanisolvens* DSM 23964, the EFSA Panel on Biological Hazards (BIOHAZ) conducted a QPS assessment for *B. xylanisolvens* (EFSA Panel on Biological Hazards, 2014). This panel found that the published studies about *B. xylanisolvens* were not sufficient to include the organism on the QPS list, although no relevant safety concerns could be established.

The *cepA* gene in the genomic DNA of *B. xylanisolvens* DSM 23964 provides the strain with resistance to β-lactam antibiotics (Ulsemer et al., 2012b). This resistance is very common in the genus *Bacteroides* (Wexler, 2007). No mobile elements, such as conjugative transposons and plasmids, were found. The strain was also screened for 8 potential virulence genes common in other *Bacteroides* species, but none was detected, and *B. xylanisolvens* DSM 23964 showed no adhesion to Caco-2 cells in the cell culture model (Ulsemer et al., 2012b). The panel considered transfer of genes unlikely to take place due to the heat inactivation and lack of plasmids.

The panel emphasized that the body of knowledge was insufficient. *B. xylanisolvens* had no history of use in fermentation processes, and the few existing studies were limited to fermented milk products. Although, safety concerns do not seem likely, the panel found the number of published studies to be too low to definitely exclude safety issues. The pilot studies involved only small, healthy cohorts, and the administered bacterial cells were inactivated. Considering all these arguments, the panel did not recommend putting *B. xylanisolvens* on the QPS list (EFSA Panel on Biological Hazards, 2014).

#### *Bacteroides xylanisolvens* and its beneficial properties

Most bacteria found in the human intestine are anaerobic. Among the many requirements for a bacterial strain to be categorized as probiotic, an essential characteristic is survival along the gastro-intestinal tract to finally provide beneficial effects for the host. *B. xylanisolvens* DSM 23964 had a survival rate of 90% after spending 3 h in simulated gastric juice and a survival rate of 96% after spending 4 h in simulated intestinal juices (Ulsemer et al., 2012b).

Immunomodulatory properties are important attributes of probiotics. The genus *Bacteroides* achieved a higher induction of mucosal IgA production than *Lactobacillus* when co-cultured with Peyer's patches lymphocytes (Yanagibashi et al., 2009). The fermentation of dietary polysaccharides with production of SCFA by *B. xylanisolvens* is connected to health-promoting effects through lower cholesterol levels, satiety stimulation, and even an anti-carcinogenic effect (Hosseini et al., 2011).

Published studies on fermented milk products containing *B. xylanisolvens* DSM 23964 are limited to inactivated bacterial cells, but by definition, probiotics need to be alive when administered to the consumer. Moreover, despite this microorganism's high survival and ability to ferment carbohydrates, these properties are irrelevant in the context of the approved product because it contains a heat-killed strain; therefore, no survival or metabolic activity is expected.

*B. xylanisolvens* offers several potential beneficial properties. However, its inability to bind to epithelial cells *in vitro* (Ulsemer et al., 2012b) is a major disadvantage in the assessment of probiotic properties because the capability to bind to intestinal epithelial cells in the host is a key step in probiotic activity. As well, studies with living bacteria are needed to increase the chances of acceptance as a probiotic.

#### Admission of *B. xylanisolvens* as a novel food: impact on other candidates

*B. xylanisolvens* is only the fourth bacterium, after *L. mesenteroides, B. subtilis* natto, and *C. butyricum*, to be approved under Novel Food Regulation No 258/97 (European Commission, 1997b).

The positive outcome of the novel food assessment is independent from the QPS evaluation results. *B. xylanisolvens* and *C. butyricum* have not yet been included on the QPS list. It should be pointed out that during the QPS assessment, only the species itself is analyzed, and the potential food products containing the bacteria are not of interest.

Thermal inactivated bacterial form falls out of the accepted definitions of probiotics described above, because the viability of bacterial cells is an essential condition. Nevertheless, non-viable and therefore non-culturable, and immunologically active microbial cells have been reported to provide health benefits to consumers (de Almada et al., 2016) The use of killed bacteria, as long as the beneficial effect is totally or partially retained, represent and advantage when the use of live bacteria is not authorized or may be potentially harmful for some individuals such as patients with weak immune system or under inflammatory conditions (Taverniti and Guglielmetti, 2011; Tsilingiri and Rescigno, 2012). To define the use of non-viable microorganisms which, when administered in adequate amounts, confer a benefit on the consumer the term “paraprobiotic” has been proposed (Taverniti and Guglielmetti, 2011; de Almada et al., 2016), but it still is not a wide accepted and employed term by scientific community and institutions.

Several mechanistic studies have demonstrated that specific chemical compounds isolated from bacteria can induce specific immune responses (Taverniti and Guglielmetti, 2011). In these cases, the term “postbiotic” has been proposed to refer any factor resulting from the metabolic activity of a bacteria or any released bacterial molecule capable of conferring beneficial effects to the consumer in a direct or indirect way (Tsilingiri and Rescigno, 2012; de Almada et al., 2016). But, similarly with “parabiotic” concept is not an officially accepted and used term.

The acceptance of the thermal inactivated form of *B. xylanisolvens* as a novel food may pave the way for other novel microorganisms in non-viable form or novel bacterial molecules to exert positive health impacts.

### Akkermansia muciniphila

The relatively newly described species *A. muciniphila* is the first discovered taxonomic member in the genus *Akkermansia*. By June 2017, there has been no evaluation according to the QPS status, which would be performed and published by EFSA. Due to the recent description of this microorganism, it has no history of use in the food industry, so it must be treated according to the Novel Food Regulation (European Commission, 1997b).

*A. muciniphila* was discovered in 2004 during the search for new mucus-degrading bacteria in human fecal samples. The newly isolated organism was named after Antoon Akkermans, a well-respected Dutch microbiologist who has made significant contributions to the field of microbial ecology (Belzer and De Vos, 2012). This oval-shaped, Gram-negative microorganism belongs to the phylum Verrucomicrobia. *A. muciniphila* further belongs to the class Verrucomicrobiae, the order Verrucomicrobiales, and the family Verrucomicrobiaceae. *A. muciniphila* was originally classified as a strict anaerobe, but in the same way as other anaerobic gut colonizers such as *Bacteroides fragilis* and *Bifidobacterium adolescentis, A. muciniphila* can tolerate small amounts of oxygen and even can use it at low oxygen concentrations (Ouwerkerk et al., 2016b). This contrasts with some of the butyrate producing bacteria that are really strict anaerobes.

Metagenomic analysis have indicated that at least 8 additional *A. muciniphila*-related species are present in the human intestine (van Passel et al., 2011). Belzer and de Vos suggested that the genus *Akkermansia* consists of 5 distinct clades (Belzer and De Vos, 2012). Four of these 5 clades are associated with *A. muciniphila*, with sequence similarity range of 80–100%.

Nearly all investigated mammals showed *Akkermansia*-related sequences in their intestinal samples. *Akkermansia*-derived sequences are not limited to mammals but also found in other vertebrates: similar sequences were detected in zebrafish and the Burmese python (Costello et al., 2010; Roeselers et al., 2011). From the latter a new species, *Akkermansia glyciniphila* was isolated and characterized from reticulated python (Ouwerkerk et al., 2016a, 2017). These ubiquitous findings suggest that *Akkermansia* played a crucial role in the early stages of vertebrata evolution and that its presence might be related to essential functions in the intestine.

Genomic analyses revealed that the single chromosome of *A. muciniphila* has 2,176 genes with a GC content of 55.8% (Donohue and Salminen, 1996). The secretome responsible for the degradation of mucin involves 61 different proteins, representing 11% of the total protein content (Belzer and De Vos, 2012).

*Akkermansia* is a relatively new genus, so it is expected that additional species similar to *A. muciniphila* will be found in coming years as is illustrated by the discovery of *A. glyciniphila*. It obviously has no history of use in the food sector, and its potential areas of application should be explored.

Despite the short time since the discovery of *Akkermansia*, its basic characteristics are well-known. This organism is non-motile and specialized in degrading mucin. *A. muciniphila* is anaerobe and chemo-organotroph and can use mucin as its sole nitrogen, carbon and energy source (Derrien et al., 2004). The habitat of *Akkermansia* spp. is also well-documented due to its ubiquitous occurrence in the intestines of many vertebrates (Belzer and De Vos, 2012). The general properties of *A. muciniphila* are listed in Table 2.

**Table 2:** General characteristics of *Akkermansia muciniphila* -adapted from Gomez-Gallego et al. (2016).

**Phylum**	**Class**	**Order**	**Family**	**Genus**	**Species**
Verrucomicrobia	Verrucomicrobiae	Verrucomicrobiales	Verrucomicrobiaceae	*Akkermansia*	*Akkermansia muciniphila*
Type strain	MucT (ATCC BAA-835)				
Biosafety level	1				
Origin	Faecal sample from a healthy adult volunteer				
**CHARACTERISTICS**					
Morphology	Gram-negative, oval-shaped, non-motile				
Genome	Size: 2,664,102 bp, ORF's: 2,176, GC content: 55.8%				
Physiology	Chemo-organotrophic, anaerobic, mesophilic (20–40°C—optimum 37°C)				
	Capable of using mucin as energy, nitrogen, and carbon source; mucolytic in pure culture				
	Sulfate release in free form from mucin fermentation				

#### Safety aspects

*A. muciniphila* is a common inhabitant of the human intestine, accounting for 1–4% of the overall colon microbiota (Derrien et al., 2008). Currently, there are no published clinical human trials in which viable cells were administered to people. Such studies have been conducted only with animal models (Everard et al., 2013). Lagier and colleagues reported two cases in humans where *A. muciniphila* accounted for up to 80% of the total intestinal microbiota without any noticeable decline in health (Lagier et al., 2015), and there is no evidence that *A. muciniphila* is connected to any specific disease (Derrien et al., 2010).

There are general concerns because *Akkermansia* shows pathogen-like behavior. Adhesion to mucus is considered to be a crucial step during infection but is also a behavior found in several probiotics. In contrast to pathogens, *A. muciniphila*, as a mucin degrader, stays in the outer layer of the mucus and never reaches the inner layer, which is necessary for a successful infection (Gomez-Gallego et al., 2016). In addition, mucin degradation itself resembles pathogen-like behavior (Donohue and Salminen, 1996) but is regarded as a regular process in a balanced, self-renewing intestine (Gomez-Gallego et al., 2016).

Colorectal cancer patients showed a four-fold increase of *A. muciniphila* in stool samples compared to healthy subjects (Weir et al., 2013). However, patients with colorectal cancer have reduced food intake, and studies have demonstrated that fasting correlates with increased levels *of A. muciniphila* (Remely et al., 2015). In addition, colorectal cancer is related to increased cell proliferation and mucus production; therefore, it is evident that levels of the main mucus-degrading bacterium could increase in this situation (Gomez-Gallego et al., 2016).

Overall, there seem to be no specific safety concerns regarding the genus *Akkermansia* and its type strain *A. muciniphila* MucT. Potential future concerns could be eliminated through individual, case-by-case review. There are no applications so far for *A. muciniphila* and, therefore, no history of safe use, so the QPS status of this species has not yet been determined.

#### *Akkermansia muciniphila* as candidate for novel food

Currently, there are no pending applications for products containing *A. muciniphila* under Regulation (EC) 258/97 (European Commission, 1997b). *A. muciniphila* was only discovered and isolated in 2004, so comprehensive research is still needed to better understand this microorganism.

*A. muciniphila* was not used for human consumption to any “significant degree” in the EU before 15 May 1997. A food business operator interested in putting a product containing *A. muciniphila* in the market would have to submit an application according to the Novel Food Regulation. The European Commission published Recommendation (European Commission, 1997a) to give food companies the information needed for a successful application. In the case of *A. muciniphila*, there are still many unknown aspects that are needed for Novel Food application, as well as some limitations: (1) specification of the possible novel products which will contain *A. muciniphila*; (2) production processes to keep *A. muciniphila* alive in the final product to fulfill its potential probiotic properties; (3) anticipated human intake taking into account the lack of known intake patterns; (4) the lack of clinical trials in humans (toxicological and nutritional assessments have been conducted only with animal models); and (5) the lack of knowledge about the allergenic potential.

Based on the knowledge acquired so far, though, this microorganism could be very interesting for food companies and consumers. *A. muciniphila* is part of the gut microbiota in all humans and may display probiotic properties due to its contribution to the functional gastro-intestinal tract. In addition, current data indicate that a decline in *A. muciniphila* in the colon correlates with several diseases, such as obesity, type-2 diabetes, and inflammatory bowel disease (Png et al., 2010; Cani and Everard, 2014; Schneeberger et al., 2015). This reduction could be related with a decrease in mucus thickness. But the complex relation between *A. muciniphila* and the mucus layer remains unclear and the administration of *A. muciniphila* might stimulate the recovery of the mucus layer (Everard et al., 2013).

Recently, *A. muciniphila* has been demonstrated to provide beneficial effects even after heat treatment for 30 min at 70°C. The heat-inactivated bacteria could reduce fat mass development, insulin resistance, and dyslipidemia in obese and diabetic mice. Some membrane proteins showed stability and interaction with Toll-like receptor 2 even during the thermal process. This membrane protein also improved the gut barrier and provided ongoing beneficial effects after bacterial inactivation (Plovier et al., 2017). Like *B. xylanisolvens*, therefore, *A. muciniphila* may enter the food market in this non-viable form.

The link between obesity and decreased levels of *A. muciniphila* extends beyond rodents. A Swedish investigation showed that obese pre-school children have significantly lower levels of *A. muciniphila* than their normal-weight peers (Karlsson et al., 2012). Similarly, pre-school infants that used macrolide antibiotics showed a higher BMI than the non-antibiotic users and this was associated with a depletion of *A.muciniphila* (Korpela et al., 2016).

Despite the growing attention to *A. muciniphila*, much research is needed to gain more specific and age-restricted information. No randomized, double-blind, placebo-controlled human clinical trials have studied the effects of *A. muciniphila* intake. Most research has involved animals, and long-term studies on the human level are the next step to take. Despite the lack of information relevant to the European Commission's recommendation on novel food, the microorganism has quite promising prospects for future novel food applications, especially for its potential probiotic health benefits.

It is important to point out that *A. muciniphila* has been detected in breast tissue (Urbaniak et al., 2014) and then has been consumed by humans via breast milk (Collado et al., 2012). The question, therefore, arises whether it is necessary to prove the safety of an organism that is already part of nutrition in the early stages of life. However, the levels of viable cells in breast milk might be much lower than those added to food products. Safety concern may arise due not to the nature of *A. muciniphila* but to the levels used in food and supplements. However, assuming around 10^13^ bacteria to be present in the human intestine, an average healthy adult may carry over 10^11^ *Akkermansia* in its colon. Further discussions are needed, but for such specific cases, European legislation needs to be modified in the future. Placement of *A. muciniphila* on the QPS list under the given conditions may be complicated because a history of safe use cannot be established.

### Fructophilic lactic acid bacteria: new members of the family

#### Characteristics

Long before Orla-Jensen described the group of lactic acid bacteria (LAB) in 1919, humankind benefited from their positive attributes in various food production processes (von Wright, 2012). LAB are involved in various industrial fermentation processes, such as fermented milk products and sausages. Along with fermented food production, the growing sector of probiotics represents the biggest operational area for LAB.

Fructophilic lactic acid bacteria (FLAB) constitute a subgroup in the diverse LAB order. FLAB preferably use fructose as a substrate. They show poor growth on glucose, and external electron acceptors (e.g., pyruvate, oxygen, and fructose) dramatically enhance their growth. FLAB, therefore, inhabit fructose-rich niches: flowers, fruits, fermented foods, such as wine and cocoa beans, and even the gastro-intestinal tracts of insects harbor these microorganisms (Endo, 2012). The FLAB group is divided into the genera *Fructobacillus* (its type species: *F*. *fructosus*) and *Lactobacillus* (Endo et al., 2009).

Four *Fructobacillus* species formerly were members of the genus *Leuconostoc*, but recent analyses suggested that their unique characteristics and phylogenetic positions supported reclassification of this bacterial group (Endo and Okada, 2008). *Fructobacillus* shows a clear preference for fructose over glucose (Endo and Okada, 2008). Several findings related to metabolism, genome size, and gene losses suggest reductive evolution in *Fructobacillus* genus compared to *Leuconostoc*, providing strong support for the reclassification and renaming of *Fructobacillus* (Endo et al., 2015).

The number of species in FLAB subgroup is growing in both genera, *Fructobacillus* and *Lactobacilllus*. Fructobacillus consists of five species: *F*. *fructosus, F. pseudoficulneus, F. ficulneus, F. durionis*, and *F. tropaeoli* (Endo et al., 2015). *F. fructosus* and *F. pseudoficulneus* are the most frequently detected species in natural sources. These microorganisms have been isolated from figs, bananas, flowers, the honeybee gut, wine, and even taberna, a traditional beverage in Southern Mexico (Alcantara-Hernandez et al., 2010; Endo, 2012). The FLAB members in the genus *Lactobacillus* are *L. kunkeei, L. apinorum*, and *L. florum* (Neveling et al., 2012). *L. kunkeei* was originally isolated from wine and characterized as obligately FLAB (Endo et al., 2012). Interestingly, this microorganism is the major component in the gut microbiota of honeybees (Endo and Salminen, 2013). *L. apinorum* is a recently described species from honeybees, and its fructophilic characteristic were recently reported (Maeno et al., 2017). These two lactobacilli share 98.9% sequence similarity based on 16S rRNA gene sequences. The genomic characteristics of the two species, especially the gene reduction system, are similar to *Fructobacillus* spp. but not to other members of lactobacilli, suggesting that fructose-richness induced an environment-specific gene reduction in phylogenetically distant microorganisms (Maeno et al., 2016). *L. florum* was formerly the only facultatively FLAB. It is differentiated from the obligately FLAB based on the growth ratio on glucose. *L. florum* grows more slowly on glucose than fructose. Like obligately FLAB, electron acceptors enhance growth on glucose.

#### Practical impact and safety

Although, FLAB were only recently discovered, this group of microorganisms might have great potential for the food industry. LAB are generally considered to be safe and have been involved in human food production and nutrition since ancient times (von Wright, 2012). These properties might be extended to FLAB. Recent investigations detected the presence of FLAB in many different food items. For example, several *Fructobacillus* spp. have been observed during the spontaneous cocoa bean fermentation process (Lefeber et al., 2011; Papalexandratou et al., 2011), and *L. florum* has been found in grapes and wine, possibly contributing important properties from an oenological perspective (Mtshali et al., 2012).

The presence of FLAB in the gastro-intestinal tracts of bees, giant ants, tropical fruit flies, and bumblebees indicate possible probiotic characteristics (Endo, 2012), although FLAB has not been detected in vertebrates' intestines (Endo and Salminen, 2013). Bee commensal *L. kunkeei* is considered to be a candidate for honeybee probiotics and paratransgenesis. There is no clinical evidence regarding the pathogenicity of FLAB, which might be an indicator of potential safety. However, this possibility should be treated cautiously because consumers' level of exposure to this bacterial group through food is not well known. Recent studies have suggested that administration of heat-killed *L. kunkeei* YB38 has potential beneficial properties for human health, including increased bowel movements and enhanced immunoglobulin A production (Asama et al., 2015, 2016). As in the case of *B. xylanisolvens*, therefore, *L. kunkeei* might enter the food market in thermal-treated form.

### Faecalibacterium prausnitzii

*Fusobacterium prausnitzii* is the sole member of the genus *Faecalibacterium* and a commensal bacterium of the human gut microbiota (Miquel et al., 2013). This Gram-positive, obligately anaerobe microorganism belongs to class Clostridia. It accounts for 3–5% of total fecal bacteria and, therefore, is a predominant species in human feces (Breyner et al., 2017). *F. prausnitzii* is a non-motile, non-spore-forming bacterium and extremely sensitive to oxygen (Foditsch et al., 2014). *F. prausnitzii* was initially classified as *F. prausnitzii*, but the sequence of the 16s rRNA gene established that it is only distantly related to *Fusobacterium* and more closely related to members of *Clostridium* cluster IV (Miquel et al., 2013). The general properties of *F. prausnitzii* are listed in Table 3.

**Table 3:** General characteristics of *Faecalibacterium prausnitzii*.

**Phylum**	**Class**	**Order**	**Family**	**Genus**	**Species**
Firmicutes	Clostridia	Clostridiales	Clostridiaceae	*Faecalibacterium*	*Faecalibacterium prausnitzii*
Type strain	ATCC 27768				
Biosafety level	1				
Origin	Faecal sample from a healthy adult volunteer				
**CHARACTERISTICS**					
Morphology	Gram-positive, obligately anaerobe, oval-shaped, non-motile, non-spore-forming				
Genome	Size: 3.05 Mb, ORF's: 2,745, GC content: 56.4%				
Physiology	Chemo-organotrophic, anaerobic, mesophilic (optimum 37°C), pH for growth ranges between 5.7 and 6.7				
	Butyrate, D-lactate, and formate production during glucose fermentation				
	Can utilize pectin, uronic acid, and host-derived substrates for growth				

*F. prausnitzii* can digest dietary fibers producing mostly butyrate, along with other SCFA (Miquel et al., 2013), and has been shown to respond to prebiotic supplementation using a mixed chain length fructan supplement and pectin (Scott et al., 2015). This microbe may play a crucial role in human health because changes in levels of *F. prausnitzii* have been associated with several gastrointestinal diseases, such as Crohn's disease, and depressive disorders (Miquel et al., 2013; Jiang et al., 2015). The microorganism also displays beneficial anti-inflammatory effects on the host, suggesting that it may be used to counterbalance the dysbiosis linked to certain diseases (Sokol et al., 2008; Jiang et al., 2015; Miquel et al., 2015). Butyrate has several beneficial effects on the host, including provision of an energy source for epithelial cells, induction of colonic regulatory T cells, induction of apoptosis in human colonic carcinoma cells, inhibition of inflammatory responses in intestinal biopsy specimens, and improvement of metabolic syndrome. Moreover, recent investigations have revealed possible contributions to protective mechanisms, such as self-defense against inflammatory reactions. *F. prausnitzii* has been involved in the inhibition of pro-inflammatory cytokines and the secretion of bioactive molecules which lead to blockage of the NF-κB pathway, showing protective effects in chemically induced colitis mouse models (Breyner et al., 2017). Further research with *Faecalibacterium* could increase knowledge about its probiotic effects and may lead to its future use in the food industry.

The successful use of *F. prausnitzii* as probiotic most likely will depend on the administration method. Studies related to bacterial resistance to gastric pH *in vitro* recommend oral administration of *F. prausnitzii* after feeding to avoid low gastric pH (Breyner et al., 2017). Moreover, cultivating *F. prausnitzii* is difficult even in anaerobic conditions (Miquel et al., 2013).

The inclusion of *Faecalibacterium* on the QPS list might be difficult due to its lack of a history of safe use and to the multiple antibiotic resistance genes observed in *Faecalibacterium* sp. isolates which could contribute to resistance dissemination (Breyner et al., 2017). In addition, full toxicology assays and characterization of the strain are still needed for regulatory approval (Thomas et al., 2015).

## Conclusions and outlook

### Remarks on the candidates

A brief evaluation of the benefits and drawbacks of the new microbial candidates based on existing knowledge is given in Table 4. Novel microbes, including many not yet assessed in humans, are increasingly proposed as potential probiotics and assessed as novel food. The introduction of these new species further challenges the traditional selection criteria because the question of their safety and efficacy arises in an untraditional manner, with no previous experience in exposure to these bacteria or their use in foods or supplements. Most probiotics belong to well-known microbial groups (lactobacilli and bifidobacteria) with long histories of safe use, which facilitates preliminary evaluation of their safety and functionality based on the available body of knowledge about these groups. However, some newly proposed probiotics were first described, cultured, and isolated during the past decade and thus present a challenge to both scientific and regulatory frontiers. Nevertheless, *A. muciniphila*, FLAB, and *F. prausnitzii* are regarded as promising candidates. The many proven and potential probiotic properties provide good opportunities for future food authorizations, but their safety in human trials still must be demonstrated.

**Table 4:** Examples of potential future probiotics suggested for human use and/or assessed as novel foods in the European Union.

**Bacteria species/strain**	**Safety assessment in European Union**	**Expected impact on humans**	**Effects (*in vitro*)**	**Effects *(in vivo*)**	**References**
*Clostridium butyricum/ Clostridium butyricum* MIYAIRI 588 *Clostridium butyricum* CGMCC0313.1	Yes	Probiotic effects suggested based on Japanese studies	Butyrate production Antitumor effect Reduction on lipogenesis	SCFAs production Prevention of antibiotic-associated diarrhea (combined with *Bifidobacterium*) Protective effect on gastric ulcers Modulation of lipid profile, insulin resistance and colon homeostasis	Nakanishi et al., 2003; Shinnoh et al., 2013; Zhao et al., 2014; Wang et al., 2015; Shang et al., 2016
*Bacteroides xylanisolvens/ Bacteroides xylanisolvens* DSM 23964 *Bacteroides xylanisolvens* XB1A	Yes, but only in the pasteurized form without any viable microbes in the product	Immune protection effects suggested	Dietary fiber degradation and fermentation	Immune modulation Toxicity and safety assessment Tolerance to a regular oral intake assessment	Mirande et al., 2010; Ulsemer et al., 2012a,b,c, 2016; Li et al., 2016
*Akkermansia muciniphila*	Potential evaluation models suggested Novel—no EFSA evaluation available yet	Weight control, control of low-grade inflammation and prevention of type 2 diabetes	Adherence to the intestinal mucosa and effect on enterocytes	Improvement of glucose tolerance and attenuation of adipose tissue inflammation Reduction of fat-mass gain Regulation of gut inflammation, gut barrier, and gut peptide secretion Reduction of metabolic endotoxemia Attenuation of atherosclerotic lesions	Donohue and Salminen, 1996; Everard et al., 2013; Reunanen et al., 2015; Schneeberger et al., 2015; Li et al., 2016; Yassour et al., 2016
Fructophilic lactic acid bacteria: *Lactobacillus kunkeei Fructobacillus fructosus Lactobacillus florum*	Novel—no evaluation available	Selective fermentation of fructose Production of antibacterial compounds	Antibacterial activity Fructophilic properties	Reduction of microbial pathogens	Endo et al., 2009, 2010; Endo, 2012; Endo and Salminen, 2013
*Faecalibacterium prausnitzii/ Faecalibacterium prausnitzii* A2-165	Novel—no evaluation available	Beneficial anti-inflammatory effects suggested Butyric acid production	Production of anti-inflammatory compounds Inhibition of IL-17 release in human monocytes Butyrate production	Increase of plasma anti-Th17 cytokines and suppression of IL-17 levels in both plasma and colonic mucosa Normalization of altered colonic permeability Protective effect against colitis	Miquel et al., 2013; Zhang et al., 2013; Laval et al., 2015; Ulluwishewa et al., 2015; Quevrain et al., 2016

*The table includes some selected examples of reported positive health effects based on in vitro and in vivo studies*.

In the context to the definition of probiotics, Plovier and colleagues made an especially interesting observation: even the pasteurized and non-replicating *Akkermansia* cells were capable of providing beneficial effects. Decreased fat-mass development, dyslipidaemia, and insulin resistance were demonstrated in obese and diabetic mice (Everard et al., 2013). This new insight might have crucial relevance and allow future authorizations because nonviable cells pose rise fewer concerns than living bacteria. Nonviable bacteria are more easily deemed safe in novel food evaluation, albeit usually in case-by-case decisions. In comparison to *B. xylanisolvens*, some information about *A. muciniphila* is still lacking. Due to the absence of practical applications, specification of the novel food and its production process and nutritional information has not yet been performed. However, there is information available about its taxonomy, culturing methods, pathogenic and toxicological nature, and nutritional assessment data in animal models (Donohue and Salminen, 1996). A major key for the approval of novel food is a sufficient number of clinical trials in humans, but *A. muciniphila* lacks enough appropriate human studies. Randomized, double-blind, placebo-controlled clinical trials, dose-response studies, and toxicological studies are still needed to, for example, establish the appropriate number of bacteria to be administered and the right matrix to provide probiotic properties. If the missing data become available, though, *A. muciniphila* has similar chances of acceptance as a novel food as the recently officially accepted *B. xylanisolvens*. Notably the EFSA positively assessed *B. xylanisolvens* although the applicant supplied only two human studies.

FLAB were also discovered and described recently, so it is challenging to predict how they might be accepted as part of future food products. Further research is needed to understand, for example, what differentiates this bacterium from traditional LAB and its exact relevance to the production of fermented foods. Recent studies revealed that heat-killed *L. kunkeei* possess beneficial properties, such as increased bowel movement and enhanced immunoglobulin A production (Asama et al., 2015, 2016). Their special relation to LAB, a very widely used group in the food industry, may lead to inclusion on the QPS list and novel food authorization in the future.

The growing amount of evidence supporting that the modulation of *F. prausnitzii* levels using prebiotics, probiotics, and symbiotics might have prophylactic or therapeutic applications in human health makes this bacterium interesting from the perspective of novel foods. Factors such as sensitivity to oxygen, gastric pH, and bile salts and industrial production for probiotic use need to be optimized, and toxicological assays and antibiotic resistance tests should be carefully conducted before clinical trials in humans.

The potential candidates for novel foods discussed as examples in the present work represent only a small fraction of the potential bacteria that may be included in novel foods in the future. Other potential candidates are *Eubacterium hallii* and bacteria in the genus *Roseburia*. *E. hallii* is a common inhabitant of the human gut microbiota and is known for its versatile utilization of different carbon sources. *E. hallii* can produce butyrate from lactate, acetate, and glucose and is one of the first butyrate producers in the infant gut (Pham et al., 2016). In addition, a recent study showed the impact of *E. hallii* in treating insulin resistance in a mouse model (Udayappan et al., 2016). Its early appearance in the human intestine and production of essential SCFA may enable future novel food applications in the food industry.

The genus *Roseburia* consists of five species which are Gram-positive, obligately anaerobic bacteria and motile due to the presence of subterminal flagella (Tamanai-Shacoori et al., 2017). *Roseburia* spp. are a dominant intestinal bacterial species, accounting for 2–15% of the total human gut microbiota that produce SCFA (Dostal et al., 2015). Considering the production of essential SCFA and the high levels of *Roseburia* spp. in the human microbiota, members of this genus may have great potential as future novel probiotics.

### Remarks on regulatory aspects

Ongoing revisions of the regulations on novel food, along with the continuous updating of the QPS list, will accelerate novel food authorization and biological agent safety assessment. Both processes support the work of the EFSA and access to novel products in the food market and ensure the safety of the European consumer. The lengthy process to revise the novel food regulations has demonstrated the difficulty of changing legislation on the European level. Most decisions need to be unanimous, and it is challenging to please all member states. Although, these developments are remarkable, the EU authorization of novel food still seems more comprehensive than the GRAS concept in the United States. The precautionary principle is a fundamental pillar of environmental, food, and health policies in the EU. Prevention of any possible harm or danger to the consumer is the most important priority, especially for marketing of food containing potentially harmful bacteria. The elimination of any possible health threat, therefore, is essential before a product is allowed to enter the European market. The European consumer is the most relevant player in European legislation although companies understandably complain about such strict conditions.

Regulation (EU) 2015/2283 on novel foods (European Commission, 2015), which will come into force on January 1, 2018, is aimed at facilitating the authorization of novel food. The EFSA is responsible for the scientific risk assessment, ensuring the shift from an individual assessment by the national competent authorities to a generic assessment. The new regulation also directs the EFSA to deliver scientific opinions within 9 months after receiving all valid applications. This mandate represents enormous time savings and a significant decrease in the financial costs for food companies submitting applications. The newly introduced data protection for approved novel food authorization will also help companies successfully compete in the food market.

## Author contributions

WK, SS, and CG designed the study; TB performed the bibliographical review; TB and CG drafting the manuscript; AE, GV, MG, Wd, WK, and SS contributed with the latest achievements in their field of expertise. AE, MG, GV, Wd, WK, SS, and CG interpreted the main points related with EU legislation discussed in the review for each novel bacteria. AE, MG, GV, Wd, WK, SS, and CG discussed the final version and revising them critically. All the authors give the final approval of the version to be published.

### Conflict of interest statement

The authors declare that the research was conducted in the absence of any commercial or financial relationships that could be construed as a potential conflict of interest.
